# Tris(2,2′-bipyridine-κ^2^ *N*,*N*′)cobalt(III) tris­(oxalato-κ^2^ *O* ^1^,*O* ^2^)ferrate(III) mono­hydrate

**DOI:** 10.1107/S1600536812003224

**Published:** 2012-02-04

**Authors:** Eduard N. Chygorin, Svitlana R. Petrusenko, Volodymyr N. Kokozay, Irina V. Omelchenko, Oleg V. Shishkin

**Affiliations:** a Department of Inorganic Chemistry, Taras Shevchenko National University of Kyiv, 64 Volodymyrs’ka Street, Kyiv 01601, Ukraine; b STC ‘Institute for Single Crystals’, National Academy of Sciences of Ukraine, 60 Lenina Avenue, Kharkiv 61001, Ukraine

## Abstract

The title compound, [Co(C_10_H_8_N_2_)_3_][Fe(C_2_O_4_)_3_]·H_2_O, con­sists of two discrete tris­(chelate) metal ions (Co^III^N_6_ and Fe^III^O_6_ chromophores) and a water mol­ecule. The structure is highly symmetrical; the Co^III^ and Fe^III^ ions occupy positions with site symmetry 3.2. The coordination polyhedra of the metal atoms have a nearly octa­hedral geometry with noticeable trigonal distortions. The Co—N and Fe—O bond lengths are equal by symmetry, *viz.* 1.981 (2) and 1.998 (4) Å, respectively. The cations and anions are arranged alternately along their threefold rotation axes parallel to [001], forming chains that are packed in a hexa­gonal manner. The water mol­ecules occupy voids between the chains. The crystal under investigation was an inversion twin.

## Related literature

For general background to direct synthesis, see: Makhankova (2011 ▶). For bond-valance sum calculation, see: Brown & Altermatt (1985 ▶) (http://www.iucr.org/resources/data/datasets/bond-valence-parameters). For related structures, see: Chygorin *et al.* (2010 ▶); Coronado *et al.* (2000 ▶); Devi *et al.* (2003 ▶); Jun & Zhang (2010 ▶); Yanagi *et al.* (1981 ▶); Zhang *et al.* (2009 ▶). For measuring of trigonal distortion angles, see: Muetterties & Guggenberger (1974 ▶). 
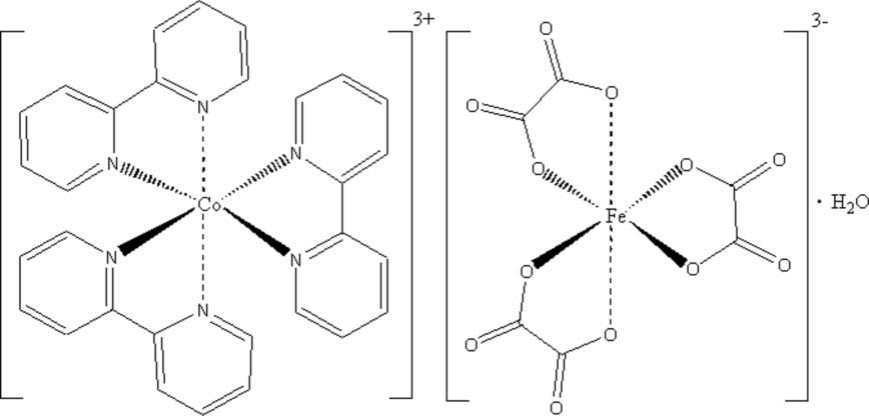


## Experimental

### 

#### Crystal data

[Co(C_10_H_8_N_2_)_3_][Fe(C_2_O_4_)_3_]·H_2_O
*M* *_r_* = 865.42
Hexagonal, 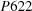

*a* = 13.056 (2) Å
*c* = 12.480 (3) Å
*V* = 1842.3 (7) Å^3^
*Z* = 2
Mo *K*α radiation
μ = 0.92 mm^−1^
*T* = 293 K
0.60 × 0.40 × 0.20 mm

#### Data collection

Oxford Diffraction Xcalibur/Sapphire3 diffractometer
Absorption correction: multi-scan (*CrysAlis RED*; Oxford Diffraction, 2009 ▶) *T*_min_ = 0.608, *T*_max_ = 0.838
17786 measured reflections
1807 independent reflections
1393 reflections with *I* > 2σ(*I*)
*R*_int_ = 0.070

#### Refinement

*R*[*F* ^2^ > 2σ(*F* ^2^)] = 0.063
*wR*(*F* ^2^) = 0.155
*S* = 1.04
1807 reflections
90 parameters
H-atom parameters constrained
Δρ_max_ = 0.36 e Å^−3^
Δρ_min_ = −0.83 e Å^−3^
Absolute structure: Flack (1983 ▶), 699 Friedel pairs
Flack parameter: 0.57 (3)

### 

Data collection: *CrysAlis CCD* (Oxford Diffraction, 2009 ▶); cell refinement: *CrysAlis RED* (Oxford Diffraction, 2009 ▶); data reduction: *CrysAlis RED*; program(s) used to solve structure: *SHELXTL* (Sheldrick, 2008 ▶); program(s) used to refine structure: *SHELXTL* and *PLATON* (Spek, 2009 ▶); molecular graphics: *SHELXTL*; software used to prepare material for publication: *publCIF* (Westrip, 2010 ▶).

## Supplementary Material

Crystal structure: contains datablock(s) I, global. DOI: 10.1107/S1600536812003224/br2188sup1.cif

Structure factors: contains datablock(s) I. DOI: 10.1107/S1600536812003224/br2188Isup2.hkl

Additional supplementary materials:  crystallographic information; 3D view; checkCIF report

## Figures and Tables

**Fig. 1.**
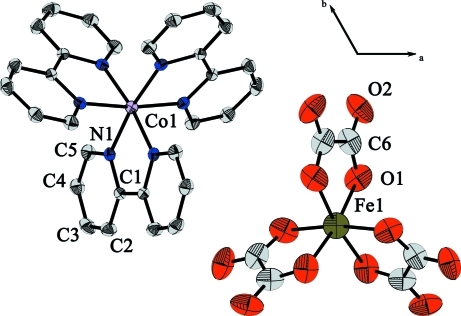
The stucture of [Co(bipy)3][Fe(Ox)3].H2O with atom labels and 30% probability displacement ellipsoids. The H atoms were omitted.

**Fig. 2.**
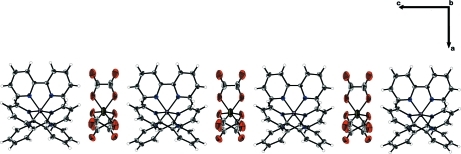
The packing of the title compound showing the linear arrangment of the complex cations and anions along the c-axis.

**Fig. 3.**
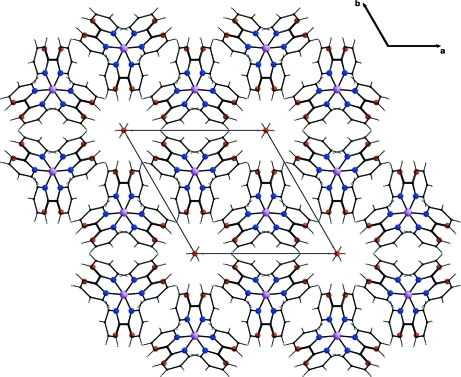
The packing of the title compound demonstrating hexagonal arrangment of the cation-anion chains and water molecules occupying voids in the interchain channels. The hydrogem atoms of water molecules are disordered into three positions.

## supplementary crystallographic information

### Comment

Developing the direct synthesis approach (Makhankova, 2011) we have investigated the following system:

Co – (NH_4_)_3_[Fe(Ox)_3_]^.^3H_2_O – bipy – 2 en^.^2HCl – CH_3_CN (in open air), where bipy = 2,2'-bipyridine, Ox = oxalate anion, en = ethylenediamine, aiming to prepare heterometallic (Co/Fe) mixed-ligand (bipy/en) complex. A pale pink precepitate that formed as a result of the reaction turned out to be a mixture with traces of undissolved cobalt powder. Recrystallization of the mixture from hot water allowed to isolate little amount of crystals of a new complex, [Co(bipy)_3_][Fe(Ox)_3_]^.^H_2_O. Structure of the complex was determined by X-ray diffraction analysis.

The crystal structure of the title compound is highly symmetrical with two sets of complex ions. The asymmetric unit contains one sixth of a [Co(bipy)_3_]^3+^ cation and one sixth of a [Fe(Ox)_3_]^3-^ anion (Fig. 1) with metal centers occupying positions with site symmetry 3.2. There are two sets of complex ions in the unit cell. All metal atoms are six coordinated (CoN_6_ and FeO_6_ chromophores). The Fe–O bond lengths are equal of 2.000 (4) Å and are typical for [Fe(Ox)_3_]^3-^ (Chygorin *et al.*, 2010, Coronado *et al.*, 2000, Zhang *et al.*, 2009) while the Co–N bond length value of 1.980 (2) Å is much larger than observed previously in [Co(bipy)_3_]^3+^ fragments (1.89 – 1.96 Å) (Devi *et al.*, 2003, Jun & Zhang, 2010, Yanagi *et al.*, 1981). Due to the rigidity of the bidentate bipyridine and oxalate ligand molecules both the cations and anions show a trigonal structure distortion *O_h_*(*D*_3_*_d_*) →*D*_3_*_h_* which should be recognized by the *cis* angles ranging from 81.92 (14)° to 92.87 (14)° for N–Co–N and from 80.7 (2)° to 94.6 (3)° for O–Fe–O. *Trans* N–Co–N and O–Fe–O angles are of 172.44 (15)° and 170.3 (3)°, respectively. More comprehensive measure of trigonal distortion is dihedral angle criterion according to which dihedral angles should be all of 70.5° for a perfect octahedron or 3×0°, 3×120° and 6×90° for a trigonal prism (Muetterties & Guggenberger, 1974). In our case, corresponding angles sets are 3×64.5°, 3×78.9° and 6×69.6° for CoN_6_ and 3×62.1°, 3×80.7° and 6×70.0° for FeO_6_ defining polyhedra as being closer to *D_3_**_d_* octahedra.

In the crystal cobalt and iron complex ions are arranged alternately along their *C_3_*-axes parallel to [001] direction forming chains (Fig. 2) with the closest Co···Fe separation of *ca* 6.2 Å. The chains are packed in a hexagonal manner (Fig. 3) and the water molecules occupy voids inside the hexagonal channels. Hydrogen atoms of water molecules are disordered to three positions accordingly to the symmetry of the channels.

The bond valence sum analysis applied to the appropriate bond lengths leads to the +3 oxidation states for both metals: 3.22 (Co) and 3.00 (Fe) using the bond valence parameters from http://www.iucr.org/resources/data/datasets/bond-valence-parameters.

It is worth noting that the described complex is the first known crystal structure with ratio [M(bipy)_3_]^3+^:[M(Ox)_3_]^3-^ equal to 1:1.

### Experimental

Cobalt powder (0.049 g, 0.83 mmol), (NH_4_)_3_[Fe(Ox)_3_]^.^3H_2_O (0.355 g, 0.83 mmol), ethylenediamine dihydrochloride (0.221 g, 1.66 mmol), 2,2' – bipyridine (0.13 g, 0.83 mmol) and acetonitrile (15 ml) were heated to 323–333 K and stirred magnetically for 6 h resulting into a pale pink precepitate. After filtration the precepitate was recrystallized from hot water. Violet block crystals were obtained after two days. The compound is stable in air, it is sparingly soluble in water, methanol and dimethylsulfoxide.

### Refinement

All hydrogen atoms were located from difference Fourier map and refined within the riding model approximation with *U*_iso_(H)= 1.5Ueq(C) for hydrogen atoms of the water molecule, and C—H = 0.93 (1)Å and *U*_iso_(H)= 1.2Ueq(C) for aromatic hydrogen atoms. Flack parameter value (Flack, 1983) of 0.57 (3) was obtained in the final structure factor calculation for enanthiopure chiral structure, that indicates presence of the both enanthiomers in the particular crystal examined. Futher full-matrix refinement of the Flack parameter slightly improved the agreement index R (from 0.0676 to 0.0625). Content of the the major enanthiomer in the refined racemic twin structure is 57 (3)%. Several isolated electron density peaks were located during the refinement, which were believed to be a solvent molecule. Large displacement parameters were observed modeling the disordered oxygen atom. SQUEEZE procedure of *PLATON* (Spek, 2009) indicated a solvent cavity of volume 161 Å^3^ centered at (0,0,0), containing approximately 21 electron. In the final refinement, this contribution was removed from the intensity data that produced better refinement results.

### Crystal data

**Table:**

[Co(C_10_H_8_N_2_)_3_][Fe(C_2_O_4_)_3_]·H_2_O	*D*_x_ = 1.560 Mg m^−^^3^
*M**_r_* = 865.42	Mo *K*α radiation, λ = 0.71073 Å
Hexagonal, *P*622	Cell parameters from 1555 reflections
Hall symbol: P 6 2	θ = 3.1–32.0°
*a* = 13.056 (2) Å	µ = 0.92 mm^−^^1^
*c* = 12.480 (3) Å	*T* = 293 K
*V* = 1842.3 (7) Å^3^	Block, violet
*Z* = 2	0.60 × 0.40 × 0.20 mm
*F*(000) = 882	

### Data collection

**Table:**

Oxford Diffraction Xcalibur/Sapphire3 diffractometer	1807 independent reflections
Radiation source: Enhance (Mo) X-ray Source	1393 reflections with *I* > 2σ(*I*)
Graphite monochromator	*R*_int_ = 0.070
Detector resolution: 16.1827 pixels mm^-1^	θ_max_ = 30.0°, θ_min_ = 3.1°
ω scans	*h* = −18→18
Absorption correction: multi-scan (*CrysAlis RED*; Oxford Diffraction, 2009)	*k* = −18→17
*T*_min_ = 0.608, *T*_max_ = 0.838	*l* = −17→17
17786 measured reflections	

### Refinement

**Table:**

Refinement on *F*^2^	Secondary atom site location: difference Fourier map
Least-squares matrix: full	Hydrogen site location: difference Fourier map
*R*[*F*^2^ > 2σ(*F*^2^)] = 0.063	H-atom parameters constrained
*wR*(*F*^2^) = 0.155	*w* = 1/[σ^2^(*F*_o_^2^) + (0.0909*P*)^2^] where *P* = (*F*_o_^2^ + 2*F*_c_^2^)/3
*S* = 1.04	(Δ/σ)_max_ < 0.001
1807 reflections	Δρ_max_ = 0.36 e Å^−^^3^
90 parameters	Δρ_min_ = −0.83 e Å^−^^3^
0 restraints	Absolute structure: Flack (1983), 699 Friedel pairs
40 constraints	Flack parameter: 0.57 (3)
Primary atom site location: structure-invariant direct methods	

### Special details

**Table:**

Experimental. CrysAlis RED, Oxford Diffraction Ltd., 2009. Empirical absorption correction using spherical harmonics, implemented in SCALE3 ABSPACK scaling algorithm.
Geometry. All e.s.d.'s (except the e.s.d. in the dihedral angle between two l.s. planes) are estimated using the full covariance matrix. The cell e.s.d.'s are taken into account individually in the estimation of e.s.d.'s in distances, angles and torsion angles; correlations between e.s.d.'s in cell parameters are only used when they are defined by crystal symmetry. An approximate (isotropic) treatment of cell e.s.d.'s is used for estimating e.s.d.'s involving l.s. planes.
Refinement. Refinement of *F*^2^ against ALL reflections. The weighted *R*-factor *wR* and goodness of fit *S* are based on *F*^2^, conventional *R*-factors *R* are based on *F*, with *F* set to zero for negative *F*^2^. The threshold expression of *F*^2^ > σ(*F*^2^) is used only for calculating *R*-factors(gt) *etc*. and is not relevant to the choice of reflections for refinement. *R*-factors based on *F*^2^ are statistically about twice as large as those based on *F*, and *R*- factors based on ALL data will be even larger.

### Fractional atomic coordinates and isotropic or equivalent isotropic displacement parameters (Å^2^)

**Table:**

	*x*	*y*	*z*	*U*_iso_*/*U*_eq_	Occ. (<1)
Co1	0.3333	0.6667	0.5000	0.0271 (3)	
Fe1	0.6667	0.3333	0.0000	0.0908 (6)	
N1	0.4659 (2)	0.6789 (2)	0.41309 (19)	0.0281 (5)	
C1	0.5760 (2)	0.7591 (2)	0.4502 (2)	0.0276 (6)	
O1	0.6801 (4)	0.4676 (4)	0.0877 (3)	0.1027 (14)	
O2	0.7734 (6)	0.6631 (5)	0.0909 (4)	0.1201 (18)	
C2	0.6771 (3)	0.7820 (3)	0.3950 (3)	0.0408 (8)	
H2	0.7512	0.8352	0.4226	0.049*	
C3	0.6678 (3)	0.7257 (3)	0.2986 (3)	0.0459 (8)	
H3	0.7352	0.7418	0.2600	0.055*	
C4	0.5576 (3)	0.6457 (3)	0.2611 (3)	0.0412 (8)	
H4	0.5490	0.6067	0.1965	0.049*	
C5	0.4604 (3)	0.6239 (3)	0.3201 (3)	0.0376 (7)	
H5	0.3863	0.5680	0.2946	0.045*	
C6	0.7526 (6)	0.5702 (7)	0.0527 (4)	0.0870 (17)	
O1W	1.0000	1.0000	0.4677 (4)	0.0314 (14)	
H1W	0.9438	1.0000	0.5000	0.047*	0.667

### Atomic displacement parameters (Å^2^)

**Table:**

	*U*^11^	*U*^22^	*U*^33^	*U*^12^	*U*^13^	*U*^23^
Co1	0.0289 (3)	0.0289 (3)	0.0235 (5)	0.01447 (16)	0.000	0.000
Fe1	0.1181 (10)	0.1181 (10)	0.0361 (8)	0.0591 (5)	0.000	0.000
N1	0.0325 (13)	0.0270 (12)	0.0280 (12)	0.0171 (11)	−0.0004 (10)	−0.0020 (10)
C1	0.0258 (12)	0.0267 (13)	0.0329 (16)	0.0149 (10)	0.0018 (12)	0.0013 (11)
O1	0.115 (3)	0.129 (4)	0.0414 (19)	0.045 (3)	0.028 (2)	0.003 (2)
O2	0.189 (5)	0.152 (4)	0.076 (3)	0.127 (4)	0.030 (3)	0.002 (3)
C2	0.0328 (16)	0.0451 (18)	0.0421 (19)	0.0176 (15)	0.0077 (15)	0.0008 (15)
C3	0.0467 (18)	0.060 (2)	0.043 (2)	0.0359 (18)	0.0151 (17)	0.0067 (16)
C4	0.0469 (18)	0.045 (2)	0.0368 (18)	0.0272 (16)	0.0051 (14)	−0.0044 (15)
C5	0.0422 (17)	0.0409 (17)	0.0317 (17)	0.0223 (14)	0.0002 (13)	−0.0082 (13)
C6	0.121 (5)	0.125 (5)	0.038 (3)	0.079 (4)	0.016 (3)	0.001 (3)
O1W	0.0168 (10)	0.0168 (10)	0.060 (4)	0.0084 (5)	0.000	0.000

### Geometric parameters (Å, º)

**Table:**

Co1—N1^i^	1.981 (2)	C1—C2	1.383 (4)
Co1—N1^ii^	1.981 (2)	C1—C1^iii^	1.454 (6)
Co1—N1^iii^	1.981 (2)	O1—C6	1.270 (7)
Co1—N1^iv^	1.981 (2)	O2—C6	1.201 (7)
Co1—N1	1.981 (2)	C2—C3	1.384 (5)
Co1—N1^v^	1.981 (2)	C2—H2	0.9300
Fe1—O1^vi^	1.998 (4)	C3—C4	1.370 (5)
Fe1—O1^vii^	1.998 (4)	C3—H3	0.9300
Fe1—O1^viii^	1.998 (4)	C4—C5	1.368 (4)
Fe1—O1^ix^	1.998 (4)	C4—H4	0.9300
Fe1—O1^x^	1.998 (4)	C5—H5	0.9300
Fe1—O1	1.998 (4)	C6—C6^viii^	1.566 (9)
N1—C5	1.348 (4)	O1W—O1W^xi^	0.807 (9)
N1—C1	1.368 (4)	O1W—H1W	0.8376
			
N1^i^—Co1—N1^ii^	81.59 (13)	O1^viii^—Fe1—O1	81.1 (2)
N1^i^—Co1—N1^iii^	93.20 (13)	O1^ix^—Fe1—O1	92.87 (17)
N1^ii^—Co1—N1^iii^	92.88 (9)	O1^x^—Fe1—O1	93.8 (3)
N1^i^—Co1—N1^iv^	92.88 (9)	C5—N1—C1	117.1 (3)
N1^ii^—Co1—N1^iv^	93.20 (13)	C5—N1—Co1	128.1 (2)
N1^iii^—Co1—N1^iv^	171.98 (13)	C1—N1—Co1	114.69 (19)
N1^i^—Co1—N1	92.88 (9)	N1—C1—C2	121.4 (3)
N1^ii^—Co1—N1	171.98 (13)	N1—C1—C1^iii^	114.47 (16)
N1^iii^—Co1—N1	81.59 (13)	C2—C1—C1^iii^	124.1 (2)
N1^iv^—Co1—N1	92.88 (9)	C6—O1—Fe1	115.4 (3)
N1^i^—Co1—N1^v^	171.98 (13)	C1—C2—C3	119.8 (3)
N1^ii^—Co1—N1^v^	92.88 (9)	C1—C2—H2	120.1
N1^iii^—Co1—N1^v^	92.88 (9)	C3—C2—H2	120.1
N1^iv^—Co1—N1^v^	81.59 (13)	C4—C3—C2	118.8 (3)
N1—Co1—N1^v^	93.20 (13)	C4—C3—H3	120.6
O1^vi^—Fe1—O1^vii^	81.1 (2)	C2—C3—H3	120.6
O1^vi^—Fe1—O1^viii^	93.8 (3)	C5—C4—C3	119.1 (3)
O1^vii^—Fe1—O1^viii^	92.87 (17)	C5—C4—H4	120.5
O1^vi^—Fe1—O1^ix^	92.87 (17)	C3—C4—H4	120.5
O1^vii^—Fe1—O1^ix^	93.8 (3)	N1—C5—C4	123.7 (3)
O1^viii^—Fe1—O1^ix^	171.3 (2)	N1—C5—H5	118.1
O1^vi^—Fe1—O1^x^	171.3 (2)	C4—C5—H5	118.1
O1^vii^—Fe1—O1^x^	92.87 (17)	O2—C6—O1	127.0 (5)
O1^viii^—Fe1—O1^x^	92.87 (17)	O2—C6—C6^viii^	119.0 (4)
O1^ix^—Fe1—O1^x^	81.1 (2)	O1—C6—C6^viii^	114.0 (3)
O1^vi^—Fe1—O1	92.87 (17)	O1W^xi^—O1W—H1W	61.2
O1^vii^—Fe1—O1	171.3 (2)		
			
N1^i^—Co1—N1—C5	−83.9 (2)	O1^viii^—Fe1—O1—C6	0.1 (3)
N1^iii^—Co1—N1—C5	−176.7 (3)	O1^ix^—Fe1—O1—C6	−173.5 (4)
N1^iv^—Co1—N1—C5	9.1 (3)	O1^x^—Fe1—O1—C6	−92.2 (4)
N1^v^—Co1—N1—C5	90.9 (3)	N1—C1—C2—C3	1.8 (5)
N1^i^—Co1—N1—C1	91.8 (2)	C1^iii^—C1—C2—C3	−177.6 (3)
N1^iii^—Co1—N1—C1	−1.05 (14)	C1—C2—C3—C4	−1.5 (5)
N1^iv^—Co1—N1—C1	−175.22 (19)	C2—C3—C4—C5	−0.1 (5)
N1^v^—Co1—N1—C1	−93.5 (2)	C1—N1—C5—C4	−1.3 (5)
C5—N1—C1—C2	−0.4 (4)	Co1—N1—C5—C4	174.3 (2)
Co1—N1—C1—C2	−176.6 (2)	C3—C4—C5—N1	1.6 (5)
C5—N1—C1—C1^iii^	179.0 (3)	Fe1—O1—C6—O2	178.4 (5)
Co1—N1—C1—C1^iii^	2.9 (4)	Fe1—O1—C6—C6^viii^	−0.2 (8)
O1^vi^—Fe1—O1—C6	93.5 (5)		

Symmetry codes: (i) −*y*+1, *x*−*y*+1, *z*; (ii) −*x*+*y*, *y*, −*z*+1; (iii) *x*, *x*−*y*+1, −*z*+1; (iv) −*x*+*y*, −*x*+1, *z*; (v) −*y*+1, −*x*+1, −*z*+1; (vi) −*x*+*y*+1, −*x*+1, *z*; (vii) *x*, *x*−*y*, −*z*; (viii) −*x*+*y*+1, *y*, −*z*; (ix) −*y*+1, *x*−*y*, *z*; (x) −*y*+1, −*x*+1, −*z*; (xi) −*y*+2, −*x*+2, −*z*+1.
